# Very high prevalence of osteosarcopenia in hip fracture patients: risk and protective factors

**DOI:** 10.1186/s13018-025-05828-7

**Published:** 2025-04-28

**Authors:** Ronald Man Yeung Wong, Pui Yan Wong, Wai Wang Chau, Chaoran Liu, Ning Zhang, Wing Hoi Cheung

**Affiliations:** Department of Orthopaedics & Traumatology, The Chinese University of Hong Kong, Hong Kong SAR, China

**Keywords:** Hip fracture, Osteosarcopenia, Fracture liaison service, Osteoporotic fracture

## Abstract

**Background:**

Hip fractures are one of the most serious forms of osteoporotic fractures. Osteosarcopenia is a growing geriatric giant with increased risk of falls, fractures, disability and mortality. The objective of this cross-sectional study was to determine the prevalence and risk factors of osteosarcopenia amongst hip fracture patients.

**Methods:**

All patients received a dual energy x-ray absorptiometry (DXA) scan for diagnosis of osteopenia and osteoporosis. For sarcopenia assessment, patients received a bioimpedance analysis (BIA) measurement, handgrip strength and 5-time chair stand test. Osteosarcopenia was defined with the presence of osteopenia/osteoporosis and sarcopenia. Risk factors for osteosarcopenia were analysed using logistic regression.

**Results:**

A total of 342 hip fracture patients (*n* = 342) were recruited. Sarcopenia was present in 286 hip fracture patients (83.6%). 335 hip fracture patients (97.95%) had osteopenia/osteoporosis. Osteosarcopenia was present in 281 hip fracture patients (82.2%). For osteosarcopenia, patients with body mass index (BMI) < 23 kg/m^2^ were 4.33 (2.35 to 7.95; *p* < 0.001) times more likely to have osteosarcopenia regardless of age and gender. Males were 3.24 (1.38 to 7.58; *p* = 0.007) more likely to have osteosarcopenia regardless of age and BMI group.

**Conclusions:**

Our study had shown a very high prevalence of osteosarcopenia amongst hip fracture patients, especially in male patients, and identified associated risk and protective factors. Given the potential clinical implications, we would recommend that in addition to bone mineral density assessment, routine sarcopenia assessment should also be incorporated into Fracture Liaison Services. Further research should be conducted on optimal body weight and BMI, and as to why male patients have more likelihood of sarcopenia.

## Background

Hip fractures are one of the most serious forms of osteoporotic fractures and with the aging population, it is projected by 2050 that there will be 6.3 million occurring worldwide [1–3]. It is well established that hip fractures should be operated in a timely manner [4, 5]. The main purpose is for early mobilization and recovery to reduce mortality and prevent medical complications which can be proven fatal [6]. The worldwide mortality rate is approximately 10% at 1 month, and reaches 36% at 1 year, and for those patients that survive, the risk of pain and disability is very high [7]. However, despite aggressive intervention and rehabilitation, 4.5 million people are disabled every year from hip fractures and it is currently ranked top 10 amongst global disabilities [6].

Sarcopenia is the progressive and generalized musculoskeletal disorder that leads to accelerated loss of muscle mass and function [8]. Sarcopenia is characterised by reductions in muscle fibre size and number, which could be due to alterations in the central and peripheral nervous system, hormonal factors changes, immunological factors and lifestyle as well [9]. It is well established that sarcopenia significantly increases the risk of falls, frailty, functional decline and mortality. A previous systematic review had shown that in patients with fragility fractures, sarcopenia could reach up to 95% in males and 64% in females [10]. Many patients experience further loss of muscle mass and strength due to poor mobility and functional recovery after a hip fracture [11]. Significant loss of skeletal muscle mass was previously reported in hip fracture patients at 1 year after surgery [12]. In fact, hip fracture patients with diagnosed sarcopenia had more than 2 times increased risk of mortality than those without. Therefore, addressing the issue of sarcopenia in hip fracture patients is of clinical importance to improve clinical outcomes.

Osteosarcopenia, defined as the presence of osteopenia/osteoporosis and sarcopenia, is a growing geriatric giant that poses a huge socioeconomic burden [13]. In fact, studies have shown that osteosarcopenia poses risk of earlier death when compared to osteoporosis and sarcopenia alone. This subset of patients has been highlighted to be more frail at higher risks of institutionalization, falls and fractures [14]. Current literature has shown limited studies focusing on osteosarcopenia in hip fracture patients. Hong Kong is a city with one of the highest life expectancies in the world and with an increasing number of elderly hip fractures [15]. Given the importance of osteosarcopenia and treatments, the objectives of this study were to (i) determine the prevalence of osteosarcopenia amongst hip fracture patients, and (ii) determine the risk factors for osteosarcopenia.

## Methods

### Study population

This was an observational study performed at the Prince of Wales Hospital, which is a tertiary academic unit in Hong Kong, China between February 2021 to May 2024 at the Fracture Liaison Service. The study was approved by the Joint Chinese University of Hong Kong – New Territories East Cluster Clinical Research Ethics Committee (CRE Ref No.: 2021.384). The study protocol is in compliance to Declaration of Helsinki and ICH-GCP. Informed consent was obtained from all subjects. The inclusion criteria in this study were (1) elderly patients aged 65 years or older (2) sustained a hip fracture, (3) low-energy trauma, (4) treated with hip fracture operation, (5) enrolled in Fracture Liaison Service. The exclusion criteria were (1) patients with open fracture, (2) pathological fracture e.g. malignancy, (3) lack of consent. As with international guidelines, for undisplaced femoral neck fractures and intertrochanteric fractures, internal fixation is performed. For displaced femoral neck fractures, a hip arthroplasty is performed. Our standard practice aims to perform the hip fracture surgery within 48 h of admission unless medically unfit as per international guidelines [6]. The data of all our hip fracture operation records are recorded in the Clinical Management System (CMS) prospectively as previously described [16]. A multidisciplinary approach is taken for hip fracture patients at our unit [17]. All patients typically receive physiotherapy with full weight-bearing the next day after hip fracture surgery as condition allows as in our previously published articles [18]. An ortho-geriatrician also identifies and treats correctable comorbidities and optimizes the medical condition of hip fracture patients [19]. Patients at the Fracture Liaison Service are typically treated for osteoporosis with anti-osteoporotic agents, calcium and vitamin D supplements at approximately 16 weeks after surgery, and this time-frame is also recommended as a key performance indicator for an effective fracture liaison service [20], also sarcopenia is assessed at the same juncture. Our recruited hip fracture patients did not have rheumatoid arthritis or glucocorticoid treatment, or secondary causes of osteoporosis, and information on previous fractures was not collected. The primary outcome of the study was to determine the prevalence of osteosarcopenia amongst hip fracture patients in a Fracture Liaison Service. The secondary outcome was to determine the risk factors associated with osteosarcopenia.

### Diagnosis for osteoporosis and osteopenia

To determine the presence of osteoporosis and osteopenia, all patients underwent a dual-energy x-ray absorptiometry (DXA) scan. The T-score was assessed at the femoral neck of the contralateral femur of the hip fracture operation and the lumbar spine. Based on current guidelines, osteoporosis and osteopenia were defined as a T score ≤ -2.5 and ≤ -1.0, respectively, at the femoral neck or lumbar spine [21]. Calibration of DXA machine was done using bone phantom, which gave an acceptable precision error of 1.31% for total hip and 0.72% for spine [22].

### Assessment for sarcopenia

To determine presence of sarcopenia, the Asian Working Group for Sarcopenia (AWGS)　2019 consensus update was used. Patients received a bioimpedance analysis (BIA) (Inbody 120, InBody Co., Ltd., Seoul, Korea) measurement to determine appendicular skeletal muscle mass (ASM) as per our previous established protocol [23]. Handgrip strength measured by the Smedley dynamometer (model EH101, Camry) to determine muscle strength and 5-time chair stand test for physical performance. A patient with low ASM/height^2^ (male: < 7.0 kg/m^2^, female: < 5.4 kg/m^2^) and either low muscle strength (male: < 28 kg, female: < 18 kg) or low physical performance (5-time chair stand test ≥ 12s) was considered to have sarcopenia [24]. Severe sarcopenia was defined with the occurrence of low ASM/height^2^ (male: < 7.0 kg/m^2^, female: < 5.4 kg/m^2^), low muscle strength (male: < 28 kg, female: < 18 kg) and low physical performance (5-time chair stand test ≥ 12s) (all 3 parameters are low) [24]. Patients were defined to have osteosarcopenia when there was concurrent presence of both osteopenia/osteoporosis and sarcopenia [13, 14].

### Statistical analysis

Subjects with any missing data were excluded from analysis. Demographic variables were summarized using mean and standard deviation for numerical variables or N (%) for categorical variables. For the demographic variables in both genders and the characteristics in the patients with different categories of muscle status, continuous data were compared using two-sample t tests or Mann-Whitney U tests for normally distributed data and non-normally distributed data, respectively. Categorical data were compared using Pearson’s chi-squared test or Fisher’s exact test. Stepwise multinomial logistic regression models were carried out to look for the predictive factors in the respective dependent variables (sarcopenia, severe sarcopenia, or osteosarcopenia). The potential predictive factors were gender (reference: female), age, body weight, body mass index (BMI), BMI < 23 kg/m^2^ (Reference: BMI < 23 kg/m^2^; based on the World Health Organization Western Pacific Region Office BMI classification for Asian adults, BMI 23–24.9 kg/m^2^ is overweight and > = 25 kg/m^2^ is obese), hip fracture type (reference: trochanteric fracture), and hip fracture operation (reference: hip screws). Statistical outcomes included odds ratio and 95% confidence interval and statistical significance in terms of p value. In the first stage, all potential predictive factors were individually entered into the regression model looking for potential factors. In the next stage onwards, those identified as potential factors were entered into the regression stepwise model. Major potential factor(s) was/were controlled by demographic variables. All statistical analysis was performed using IBM SPSS version 29 (Armonk, NY). P-value of ≤ 0.05 was considered statistically significant.

## Results

### Demographics

**Fig. 1:**
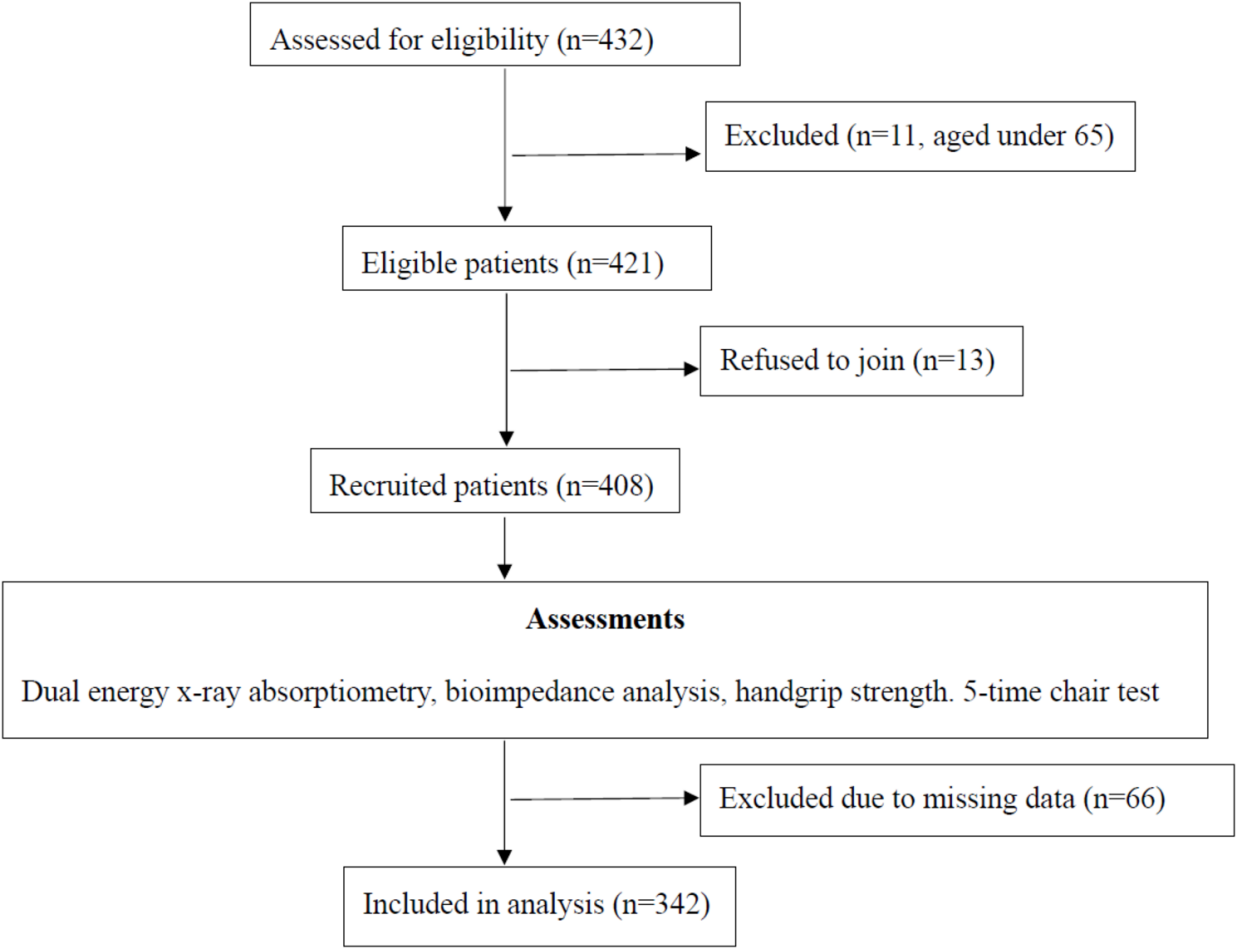
The flow diagram

A total of 342 Chinese hip fracture patients (*n* = 342) were recruited and analysed in this observational study (Fig. 1). The average age was 81.65 ± 7.63 years old, and 22.8% (*n* = 78) were male patients. The average body mass index (BMI) was 22.40 ± 3.66 kg/m^2^. A total of 158 patients (46.5%) had intertrochanteric fractures and 182 patients (53.5%) had femoral neck fractures. All patients had undergone hip fracture operation. Refer to Table 1.

**Table 1:** Demographic characteristics of the 342 hip fracture patients

Demographic characteristics	*N* = 342	Male (*n* = 78)	Female (*n* = 264)	*P*
**Gender**				
Male	78 (22.8)			
Female	264 (77.2)			
Age, year				
Mean ± SD	81.65 ± 7.63	80.29 ± 7.93	81.94 ± 7.63	0.254
Body weight, kg				
Mean ± SD	53.26 ± 10.79	61.01 ± 10.23	50.97 ± 9.86	< 0.001
BMI				
Mean ± SD	22.40 ± 3.66	22.84 ± 3.23	22.27 ± 3.77	0.099
**Overweight**				0.195
BMI ≥ 23	132 (38.6)	35 (44.9)	97 (36.7)	
BMI < 23	210 (61.4)	43 (55.1)	167 (63.3)	
**Hip fracture type**				0.081
Neck of femur	182 (53.5)	35 (44.9)	147 (56.1)	
Trochanteric	158 (46.5)	43 (55.1)	115 (43.9)	
**Hip fracture operation**				0.021
Cephalomedullary nail	158 (46.7)	40 (52.6)	118 (45.0)	
Hemiarthroplasty	104 (30.8)	18 (23.7)	86 (32.8)	
Hip Screw	60 (17.8)	10 (13.2)	50 (19.1)	
Total hip replacement	16 (4.7)	8 (10.5)	8 (3.1)	

### Diagnosis of sarcopenia

The average ASM/height^2^ was 4.90 ± 0.80 kg/m^2^. A total of 296 hip fracture patients (86.5%) failed the muscle mass test. The average handgrip strength of the patients was 16.3 ± 10.6 kg. A total of 278 hip fracture patients (81.3%) failed the muscle strength test. Physical performance was assessed by the 5-time chair stand test. The average time of 5-time chair test was 24.4 ± 12.3s. A total of 309 hip fracture patients (90.4%) failed the physical performance test. The diagnosis of sarcopenia based on the AWGS 2019 consensus update was present in 286 hip fracture patients (83.6%), of which 223 hip fracture patients had severe sarcopenia (65.2%). Compared with patients without sarcopenia, patients with sarcopenia were significantly older (*p* = 0.008) and had lower BMI (*p* < 0.001). There was also a higher percentage of sarcopenia in males than in females (*p* = 0.002). Refer to Table 2.

**Table 2:** Characteristics of participants with or without sarcopenia, osteopenia/osteoporosis and osteosarcopenia

Characteristics	No sarcopenia	Sarcopenia		No Osteopenia/Osteoporosis	Osteopenia/Osteoporosis		No Osteosarcopenia	Osteosarcopenia	
Total number	*n* = 56	*n* = 286	*P*	*n* = 7	*n* = 335	*P*	*n* = 61	*n* = 281	*P*
Age (years)	79.20 ± 6.70	82.13 ± 7.71	0.008	75.43 ± 7.32	81.78 ± 7.59	0.029	78.72 ± 6.87	82.28 ± 7.64	< 0.001
**Age group**			0.207			0.036			0.035
65-69	5 (8.9)	16 (5.6)		2 (28.6)	19 (5.7)		7 (11.5)	14 (5.0)	
70-79	22 (39.3)	88 (30.8)		3 (42.9)	107 (31.9)		24 (39.3)	86 (30.6)	
>80	29 (51.8)	182 (63.6)		2 (28.6)	209 (62.4)		30 (49.2)	181 (64.4)	
**Gender**			0.002			0.05			0.047
Male	4 (7.1)	74 (25.9)		4 (57.1)	74 (22.1)		8 (13.1)	70 (24.9)	
Female	52 (92.9)	212 (74.1)		3 (42.9)	261 (77.9)		53 (86.9)	211 (75.1)	
BMI, (kg/m^2^)	24.47 ± 4.46	21.80 ± 3.16	< 0.001	26.08 ± 5.36	22.32 ± 3.59	0.007	25.32 ± 4.43	21.77 ± 3.14	< 0.001
**BMI group**			< 0.001						
BMI < 23	18 (32.1)	192 (67.1)		3 (42.9)	207 (61.8)	0.436	21 (34.4)	189 (67.3)	< 0.001
BMI ≥ 23	38 (67.9)	94 (32.9)		4 (57.1)	128 (38.2)		40 (65.6)	92 (32.7)	
**ASM / Ht** ^**2**^ **(kg/m** ^**2**^ **)**	5.72 ± 0.91	4.73 ± 0.66	< 0.001	5.75 ± 0.75	4.88 ± 0.78	0.004	5.71 ± 0.90	4.72 ± 0.64	< 0.001
Male	6.93 ± 0.85	5.4 ± 0.58	< 0.001	5.98 ± 0.52	5.47 ± 0.69	0.144	6.46 ± 0.83	5.38 ± 0.58	< 0.001
Female	5.62 ± 0.85	4.50 ± 0.50	< 0.001	5.43 ± 1.01	4.72 ± 0.73	0.092	5.60 ± 0.86	4.50 ± 0.50	< 0.001

Data are expressed as n (%) or mean ± SD. SD, standard deviation; BMI, body mass index; ASM, appendicular skeletal muscle mass, Ht, height

For female patients (*n* = 264), the average ASM/height^2^ was 4.72 ± 0.73 kg/m^2^. A total of 220 female hip fracture patients (83.3%) failed the muscle mass test. The average handgrip strength was 14.6 ± 11.0 kg. A total of 213 female hip fracture patients (80.7%) failed the muscle strength test. The average time of 5-time chair test was 25.0 ± 12.4s. A total of 238 female hip fracture (90.2%) failed the physical performance test. The diagnosis of sarcopenia based on the AWGS 2019 consensus update was present in 212 female hip fracture patients (80.3%), of which 163 female hip fracture patients had severe sarcopenia (61.7%).

For male patients (*n* = 78), the average ASM/height^2^ was 5.50 ± 0.68 kg/m^2^. A total of 76 male hip fracture patients (97.4%) failed the muscle mass test. The average handgrip strength was 22.3 ± 6.6 kg. A total of 65 male hip fracture patients (83.3%) failed the muscle strength test. The average time of 5-time chair test was 22.7 ± 12.0s. A total of 71 male hip fracture (91.0%) failed the physical performance test. The diagnosis of sarcopenia based on the AWGS 2019 consensus update was present in 74 hip fracture patients (94.9%), of which 60 male hip fracture patients had severe sarcopenia (76.9%).

### Diagnosis of osteoporosis/osteopenia

Osteoporosis as defined by a T score ≤ -2.5 was present in 236 hip fracture patients (69.0%). There were 335 hip fracture patients (98.0%) who had osteopenia/osteoporosis as defined by a T score ≤ -1.0. Compared with patients without osteopenia/osteoporosis, patients with osteopenia/osteoporosis were significantly older (*p* = 0.029) and had lower BMI (*p* = 0.007). Refer to Table 2.

For female hip fracture patients (*n* = 264), osteopenia/osteoporosis was present in 261 (98.9%) patients, of which osteoporosis was present in 208 (78.8%) patients. For male hip fracture patients (*n* = 78), osteopenia/osteoporosis was present in 74 (94.9%) patients, of which osteoporosis was present in 26 (33.3%) patients.

### Diagnosis of osteosarcopenia

The combination of low bone density (osteopenia/osteoporosis) and sarcopenia was present in 281 hip fracture patients (82.2%). Compared with patients without osteosarcopenia, patients with osteosarcopenia were significantly older (*p* < 0.001), had lower BMI (*p* < 0.001) and there was a higher percentage of having sarcopenia in males than in females (*p* = 0.047). Refer to Table 2. For female hip fracture patients (*n* = 264), osteosarcopenia was present in 211 (79.9%) patients. For male hip fracture patients (*n* = 78), osteosarcopenia was present in 70 (89.7%) patients.

### Logistic regression analysis

Sarcopenia was 1.05 (1.01 to 1.09) and 4.31 (2.34 to 7.96) times more likely to occur with increased age (*p* = 0.013) and BMI < 23 kg/m^2^ (*p* < 0.001), respectively. Increased body weight and BMI had an odds ratio of 0.94 (0.91 to 0.96) (*p* < 0.001) and 0.76 (0.70 to 0.83) (*p* < 0.001) for sarcopenia. The odds of having sarcopenia in males to females was 4.54 (1.59 to 12.98) (*p* = 0.005). As for severe sarcopenia, increased age had 1.08 (1.05 to 1.12) (*p* < 0.001) times more likelihood of occurrence. Similarly, increased body weight and BMI had an odds ratio of 0.95 (0.93 to 0.97; *p* < 0.001) and 0.86 (0.80 to 0.91; *p* < 0.001), respectively. The odds of having severe sarcopenia in males to females was 2.07 (1.15 to 3.70) (*p* = 0.015). Further adjustment of confounding variables showed that increased age was 1.90 (1.06 to 1.13) times (*p* < 0.001) more likely to have severe sarcopenia regardless of gender and BMI group. Males were 2.96 (1.56 to 5.64) times (*p* < 0.001) more likely to have severe sarcopenia regardless of age and BMI group. Refer to Table 3.

**Table 3:** Logistic regression analysis of risk factors for sarcopenia and osteosarcopenia

	Sarcopenia				Severe sarcopenia				Osteosarcopenia			
	Unadjusted		Adjusted		Unadjusted		Adjusted		Unadjusted		Adjusted	
	OR (95% CI)	*P*	OR (95% CI)	*P*	OR (95% CI)	*P*	OR (95% CI)	*P*	OR (95% CI)	*P*	OR (95% CI)	*P*
Age (+ 1 year)	1.05 (1.01–1.09)	0.013	1.06 (1.02–1.11)	0.005	1.08 (1.05–1.12)	< 0.001	1.90 (1.06–1.13)	< 0.001	1.06 (1.02–1.10)	0.002	1.07 (1.03–1.11)	< 0.001
Gender (ref: female)	4.54 (1.59–12.98)	0.005	7.05 (2.34–21.28)	< 0.001	2.07 (1.15–3.70)	0.015	2.96 (1.56–5.64)	< 0.001	2.20 (1.00-4.85)	0.051	3.24 (1.38–7.58)	0.007
Weight (+ 1 kg)	0.94 (0.91–0.96)	< 0.001	0.90 (0.87–0.94)	< 0.001	0.95 (0.93–0.97)	< 0.001	0.93 (0.91–0.96)	< 0.001	0.93 (0.91–0.96)	< 0.001	0.91 (0.88–0.94)	< 0.001
BMI group (ref: BMI ≥ 23 kg/m^2^)	4.31 (2.34–7.96)	< 0.001	5.08 (2.67–9.64)	< 0.001	2.38 (1.51–3.76)	< 0.001	2.60 (1.59–4.24)	< 0.001	3.91 (2.18–7.02)	< 0.001	4.33 (2.35–7.95)	< 0.001

OR, odds ratio; CI, confidence interval; BMI, body mass index

For osteosarcopenia, increased age and BMI < 23 kg/m^2^ had 1.06 (1.02 to 1.10; *p* = 0.002) and 3.91 (2.18 to 7.02; *p* < 0.001) times, respectively, more likelihood of occurrence. Increased body weight and BMI had an odds ratio of 0.93 (0.91 to 0.96; *p* < 0.001) and 0.77 (0.70 to 0.83; *p* < 0.001) for osteosarcopenia. The odds of having osteosarcopenia in males to females was 2.20 (1.00 to 4.85; *p* = 0.051). Further adjustment of confounding variables showed that increased age and body weight were 1.07 (1.03 to 1.11; *p* < 0.001) and 0.91 (0.88 to 0.94; *p* < 0.001) times more likelihood of osteosarcopenia, respectively, regardless of gender. Patients with BMI < 23 kg/m^2^ were 4.33 (2.35 to 7.95; *p* < 0.001) times more likely to have osteosarcopenia regardless of age and gender. Males were 3.24 (1.38 to 7.58) times (*p* = 0.007) more likely to have osteosarcopenia regardless of age and BMI group. Refer to Table 3.

## Discussion

Osteosarcopenia is a unique syndrome defined by low bone density (osteopenia/osteoporosis) and sarcopenia. In fact, with the aging population, the entity has now become a geriatric giant. Studies have shown that osteosarcopenic patients are at higher risk of falls, fractures, disability and mortality leading to significant socioeconomic costs [14]. In our study, we identified a very high prevalence of osteosarcopenia in hip fracture patients at 82.2%. A previous study from Korea showed the prevalence of osteosarcopenia to be 28.7% in hip fracture patients [25], and another study from Italy showed the prevalence to be 65.7% [26]. The much higher prevalence identified in our study may be due to the high life expectancy in Hong Kong, which is one of the highest in the world. Furthermore, frailty is common amongst hip fracture patients, in which many would be sarcopenic [27]. Our study highlights as one of the few studies to show the prevalence of osteosarcopenia in hip fracture patients. More importantly, osteosarcopenia has been shown to be common amongst hip fracture patients and should be identified for prompt treatment.

We found a higher likelihood at 3.24 (1.38 to 7.58) times of men having osteosarcopenia after controlling confounding factors. Previous studies have shown that women may be more susceptible to osteosarcopenia in the general adult population [28]. However, a previous systematic review also showed that the prevalence of sarcopenia in fragility fracture patients was higher in males (12.4–95%) than females (18.3–64%) [10]. The limitation is that not all studies use standardised protocols to diagnose sarcopenia [29]. Possible explanation to the current findings may be that males have approximately twice as fast muscle degradation compared to women and also lose more testosterone [30]. A previous study also identified that male patients performed worse in terms of mobility after hip fracture surgery compared to females [31]. Another cross-sectional study showed that there were more males compared to females having sarcopenia in community-dwelling elderlies [32]. Further studies may be required to further verify this.

Although the treatment of osteoporosis has been well established, with potent anti-osteoporotic agents including anabolic agents [33], there is currently no Food and Drug Administration (FDA) approved drug to treat sarcopenia. Unfortunately, sarcopenia is associated with premature mortality, and therefore finding solutions to treat the disease is crucial. Hip fracture is a major public health concern and is one of the most serious forms of osteoporotic fractures, often leading to disability and poor clinical outcomes [16, 19, 31]. It is also well-known that the mortality rates can reach as high as 36% in 1 year [6]. In our study, the prevalence of sarcopenia amongst hip fracture patients was very high at 83.6%. A recent study also showed that sarcopenia increases postoperative mortality and recovery of patients in orthopaedic surgery [34]. Therefore, this further highlights that new strategies to treat these patients are warranted. Several commonly used consensus have been established with the use of common definitions including European (European Working Group on Sarcopenia in Older People), Asian (Asian Working Group on Sarcopenia) and American (Sarcopenia Definitions and Outcomes Consortium) [35]. In our study, we had used the AWGS 2019 consensus update to diagnose sarcopenia in the hip fracture patients. Lifestyle intervention with nutrition and resistance exercise are the mainstay of treatment for sarcopenia [36]. There appears to be insufficient evidence for the use of vitamin D and anabolic steroids [36]. There is still a need for ongoing clinical studies to assess and identify effective pharmacological treatments for sarcopenia. Recent preclinical and clinical studies have also shown potential of using probiotics to modulate the gut microbiota [37, 38].

Logistic regression analysis showed that age and BMI < 23 kg/m^2^ were risk factors of sarcopenia and osteosarcopenia, whilst increased body weight and BMI were protective factors. The obesity paradox has been referred to as an observation that although obesity can lead to adverse clinical outcomes including cardiovascular diseases, there may be an inverse relationship between BMI and mortality [39]. Another study showed that older adults with BMI < 25 kg/m^2^ and > 35 kg/m^2^ were at a higher risk of experiencing gait and balance problem, as well as decreased muscle strength [40]. Interestingly, a previous meta-analysis showed that obesity was associated with reduced risk of sarcopenia, but in fact, this attenuated risk is actually dependent on higher muscle mass and strength [41]. People with obesity have greater absolute muscle mass and strength compared to lean people [41]. A previous study also showed that low muscle mass and function is harmful to bone health, and therefore treating muscle health is important [42]. Fracture type and operation type were not found to be significantly different amongst hip fracture patients for sarcopenia and osteosarcopenia. This reinforces that it is mainly dependent on the patient premorbid status.

The Fracture Liaison Service is well recognized to prevent secondary fractures amongst fragility fracture patients [17]. In our study, sarcopenia was assessed on all the hip fracture patients, and it has now been recommended to incorporate this in Fracture Liaison Services [33]. With the high prevalence of osteosarcopenia in hip fracture patients, this service is an important aspect for treatment. The strengths of the study are that the AWGS 2019 consensus update to diagnose sarcopenia was used, and there was a good sample size to determine the data. To our knowledge, our study is also one of the first amongst Chinese patients, providing important data for reference and provides strong clinical implications. The limitations of the study are that this was a single-centre study, and there was no follow-up data. Furthermore, the assessment of osteosarcopenia was approximately 16 weeks after hip fracture surgery, where the muscle mass and bone mass loss can be affected, which may lead to a higher incidence of osteosarcopenia.

## Conclusions

In conclusion, our study has shown a very high prevalence of osteosarcopenia, especially in males, amongst Chinese hip fracture patients. We would recommend that routine sarcopenia assessment should be incorporated in Fracture Liaison Services given the potential clinical implications.

## Data Availability

Data is contained within the article. Individual data is unavailable due to privacy or ethical restrictions.

## Abbreviations

ASM: Appendicular Skeletal Muscle Mass
AWGS: Asian Working Group for Sarcopenia
BIA: Bioimpedance Analysis
BMI: Body Mass Index
CMS: Clinical Management System
DXA: Dual Energy X-ray Absorptiometry
FDA: Food and Drug Administration
ICH-GCP: International Conference on Harmonisation - Good Clinical Practice

## References

[1] Papadimitriou N, Tsilidis KK, Orfanos P, Benetou V, Ntzani EE, Soerjomataram I, et al. Burden of hip fracture using disability-adjusted life-years: a pooled analysis of prospective cohorts in the CHANCES consortium. Lancet Public Health. 2017;2(5):e239–46. 29253489 10.1016/S2468-2667(17)30046-4

[2] Cooper C, Cole ZA, Holroyd CR, Earl SC, Harvey NC, Dennison EM, et al. Secular trends in the incidence of hip and other osteoporotic fractures. Osteoporos Int. 2011;22(5):1277–88. 21461721 10.1007/s00198-011-1601-6 PMC3546313

[3] Gargano G, Poeta N, Oliva F, Migliorini F, Maffulli N. Zimmer natural nail and ELOS nails in Pertrochanteric fractures. J Orthop Surg Res. 2021;16(1):509. 34407829 10.1186/s13018-021-02634-9 PMC8371819

[4] Marsillo E, Pintore A, Asparago G, Oliva F, Maffulli N. Cephalomedullary nailing for reverse oblique intertrochanteric fractures 31A3 (AO/OTA). Orthop Rev (Pavia). 2022;14(6):38560. 36267220 10.52965/001c.38560 PMC9568432

[5] Quaranta M, Miranda L, Oliva F, Migliorini F, Pezzuti G, Maffulli N. Haemoglobin and transfusions in elderly patients with hip fractures: the effect of a dedicated orthogeriatrician. J Orthop Surg Res. 2021;16(1):387. 34134743 10.1186/s13018-021-02524-0 PMC8207795

[6] Bhandari M, Swiontkowski M. Management of acute hip fracture. N Engl J Med. 2017;377(21):2053–62. 29166235 10.1056/NEJMcp1611090

[7] Bhandari M, Einhorn TA, Guyatt G, Schemitsch EH, Zura RD, Sprague S, et al. Total hip arthroplasty or hemiarthroplasty for hip fracture. N Engl J Med. 2019;381(23):2199–208. 31557429 10.1056/NEJMoa1906190

[8] Cruz-Jentoft AJ, Sayer AA, Sarcopenia. Lancet. 2019;393(10191):2636–46. 10.1016/S0140-6736(19)31138-9 31171417

[9] Narici MV, Maffulli N. Sarcopenia: characteristics, mechanisms and functional significance. Br Med Bull. 2010;95:139–59. 20200012 10.1093/bmb/ldq008

[10] Wong RMY, Wong H, Zhang N, Chow SKH, Chau WW, Wang J, et al. The relationship between sarcopenia and fragility fracture-a systematic review. Osteoporos Int. 2019;30(3):541–53. 30610245 10.1007/s00198-018-04828-0

[11] Maffulli N, Aicale R. Proximal femoral fractures in the elderly: A few things to know, and some to forget. Med (Kaunas). 2022;58(10). 10.3390/medicina58101314 PMC9612001 36295475

[12] Chen YP, Kuo YJ, Hung SW, Wen TW, Chien PC, Chiang MH, et al. Loss of skeletal muscle mass can be predicted by sarcopenia and reflects poor functional recovery at one year after surgery for geriatric hip fractures. Injury. 2021;52(11):3446–52. 34404509 10.1016/j.injury.2021.08.007

[13] Kirk B, Zanker J, Duque G. Osteosarcopenia: epidemiology, diagnosis, and treatment-facts and numbers. J Cachexia Sarcopenia Muscle. 2020;11(3):609–18. 32202056 10.1002/jcsm.12567 PMC7296259

[14] Hirschfeld HP, Kinsella R, Duque G. Osteosarcopenia: where bone, muscle, and fat collide. Osteoporos Int. 2017;28(10):2781–90. 28733716 10.1007/s00198-017-4151-8

[15] Wong RMY, Ho WT, Wai LS, Li W, Chau WW, Chow KS, et al. Fragility fractures and imminent fracture risk in Hong Kong: one of the cities with longest life expectancies. Arch Osteoporos. 2019;14(1):104. 31659457 10.1007/s11657-019-0648-4

[16] Wong RMY, Ng RWK, Chau WW, Liu WH, Chow SKH, Tso CY, et al. Montreal cognitive assessment (MoCA) is highly correlated with 1-year mortality in hip fracture patients. Osteoporos Int. 2022;33(10):2185–92. 10.1007/s00198-022-06426-7 35763077

[17] Wong RMY, Law SW, Lee KB, Chow SKH, Cheung WH. Secondary prevention of fragility fractures: instrumental role of a fracture liaison service to tackle the risk of imminent fracture. Hong Kong Med J. 2019;25(3):235–42. 31182670 10.12809/hkmj187593

[18] Wong RMY, Wong PY, Liu C, Chui CS, Liu WH, Tang N, et al. Vibration therapy as an intervention for trochanteric hip fractures - A randomized double-blinded, placebo-controlled trial. J Orthop Translat. 2025;51:51–8. 39926341 10.1016/j.jot.2025.01.002 PMC11802369

[19] Wong RMY, Zu Y, Chau WW, Tso CY, Liu WH, Ng RWK, et al. High Charlson comorbidity index score is associated with early fracture-related complication for internal fixation of neck of femur fractures. Sci Rep. 2022;12(1):4749. 35306533 10.1038/s41598-022-08855-0 PMC8934361

[20] Javaid MK, Sami A, Lems W, Mitchell P, Thomas T, Singer A, et al. A patient-level key performance indicator set to measure the effectiveness of fracture liaison services and guide quality improvement: a position paper of the IOF capture the fracture working group, National osteoporosis foundation and fragility fracture network. Osteoporos Int. 2020;31(7):1193–204. 32266437 10.1007/s00198-020-05377-1 PMC7280347

[21] Kanis JA, Cooper C, Rizzoli R, Reginster JY, Scientific Advisory Board of the European Society for C., Economic Aspects of O, European guidance for the diagnosis and management of osteoporosis in postmenopausal women. Osteoporos Int. 2019;30(1):3–44. 10.1007/s00198-018-4704-5 PMC7026233 30324412

[22] Leung KS, Li CY, Tse YK, Choy TK, Leung PC, Hung VW, et al. Effects of 18-month low-magnitude high-frequency vibration on fall rate and fracture risks in 710 community elderly–a cluster-randomized controlled trial. Osteoporos Int. 2014;25(6):1785–95. 24676848 10.1007/s00198-014-2693-6

[23] Cheng KY, Chow SK, Hung VW, Wong CH, Wong RM, Tsang CS, et al. Diagnosis of sarcopenia by evaluating skeletal muscle mass by adjusted bioimpedance analysis validated with dual-energy X-ray absorptiometry. J Cachexia Sarcopenia Muscle. 2021;12(6):2163–73. 34609065 10.1002/jcsm.12825 PMC8718029

[24] Chen LK, Woo J, Assantachai P, Auyeung TW, Chou MY, Iijima K, et al. Asian working group for sarcopenia: 2019 consensus update on sarcopenia diagnosis and treatment. J Am Med Dir Assoc. 2020;21(3):300–e72. 32033882 10.1016/j.jamda.2019.12.012

[25] Yoo JI, Kim H, Ha YC, Kwon HB, Koo KH. Osteosarcopenia in patients with hip fracture is related with high mortality. J Korean Med Sci. 2018;33(4):e27. 29318794 10.3346/jkms.2018.33.e27 PMC5760812

[26] Di Monaco M, Castiglioni C, Bardesono F, Milano E, Massazza G. Sarcopenia, osteoporosis and the burden of prevalent vertebral fractures: a cross-sectional study of 350 women with hip fracture. Eur J Phys Rehabil Med. 2020;56(2):184–90. 32052946 10.23736/S1973-9087.20.05991-2

[27] Wilson D, Jackson T, Sapey E, Lord JM. Frailty and sarcopenia: the potential role of an aged immune system. Ageing Res Rev. 2017;36:1–10. 28223244 10.1016/j.arr.2017.01.006

[28] Huang T, Li C, Chen F, Xie D, Yang C, Chen Y, et al. Prevalence and risk factors of osteosarcopenia: a systematic review and meta-analysis. BMC Geriatr. 2023;23(1):369. 37322416 10.1186/s12877-023-04085-9 PMC10273636

[29] Testa G, Vescio A, Zuccala D, Petrantoni V, Amico M, Russo GI et al. Diagnosis, treatment and prevention of sarcopenia in hip fractured patients: where we are and where we are going: A systematic review. J Clin Med. 2020;9(9). 10.3390/jcm9092997 PMC7563874 32957453

[30] Lee A, McArthur C, Ioannidis G, Duque G, Adachi JD, Griffith LE, et al. Associations between osteosarcopenia and falls, fractures, and frailty in older adults: results from the Canadian longitudinal study on aging (CLSA). J Am Med Dir Assoc. 2024;25(1):167–76. e6. 37925161 10.1016/j.jamda.2023.09.027

[31] Wong RMY, Qin J, Chau WW, Tang N, Tso CY, Wong HW, et al. Prognostic factors related to ambulation deterioration after 1-year of geriatric hip fracture in a Chinese population. Sci Rep. 2021;11(1):14650. 34282186 10.1038/s41598-021-94199-0 PMC8289836

[32] Du Y, Wang X, Xie H, Zheng S, Wu X, Zhu X, et al. Sex differences in the prevalence and adverse outcomes of sarcopenia and sarcopenic obesity in community dwelling elderly in East China using the AWGS criteria. BMC Endocr Disord. 2019;19(1):109. 31653213 10.1186/s12902-019-0432-x PMC6814981

[33] Wong RMY, Cheung WH, Chow SKH, Ng RWK, Li W, Hsu AY, et al. Recommendations on the post-acute management of the osteoporotic fracture - Patients with very-high Re-fracture risk. J Orthop Translat. 2022;37:94–9. 36262963 10.1016/j.jot.2022.09.010 PMC9562437

[34] Brzeszczynski F, Brzeszczynska J, Duckworth AD, Murray IR, Simpson A, Hamilton DF. The effect of sarcopenia on outcomes following orthopaedic surgery: a systematic review. Bone Joint J. 2022;104–B(3):321–30. 35227092 10.1302/0301-620X.104B3.BJJ-2021-1052.R1

[35] Reginster JY, Beaudart C, Al-Daghri N, Avouac B, Bauer J, Bere N, et al. Update on the ESCEO recommendation for the conduct of clinical trials for drugs aiming at the treatment of sarcopenia in older adults. Aging Clin Exp Res. 2021;33(1):3–17. 32737844 10.1007/s40520-020-01663-4 PMC7897619

[36] Bauer J, Morley JE, Schols A, Ferrucci L, Cruz-Jentoft AJ, Dent E, et al. Sarcopenia: A time for action. An SCWD position paper. J Cachexia Sarcopenia Muscle. 2019;10(5):956–61. 31523937 10.1002/jcsm.12483 PMC6818450

[37] Liu C, Wong PY, Wang Q, Wong HY, Huang T, Cui C, et al. Short-chain fatty acids enhance muscle mass and function through the activation of mTOR signalling pathways in sarcopenic mice. J Cachexia Sarcopenia Muscle. 2024;15(6):2387–401. 39482890 10.1002/jcsm.13573 PMC11634463

[38] Liu C, Wong PY, Barua N, Li B, Wong HY, Zhang N, et al. From clinical to benchside: Lacticaseibacillus and Faecalibacterium are positively associated with muscle health and alleviate Age-Related muscle disorder. Aging Cell. 2025;0:e14485. 10.1111/acel.14485 PMC12073917 39829204

[39] Liu C, Cheng KY, Tong X, Cheung WH, Chow SK, Law SW, et al. The role of obesity in sarcopenia and the optimal body composition to prevent against sarcopenia and obesity. Front Endocrinol (Lausanne). 2023;14:1077255. 36936175 10.3389/fendo.2023.1077255 PMC10016224

[40] Kıskaç M, Soysal P, Smith L, Capar E, Zorlu M. What is the optimal body mass index range for older adults?? Ann Geriatr Med Res. 2022;26(1):49–57. 35368193 10.4235/agmr.22.0012 PMC8984168

[41] Liu C, Wong PY, Chung YL, Chow SK, Cheung WH, Law SW, et al. Deciphering the obesity paradox in the elderly: A systematic review and meta-analysis of sarcopenic obesity. Obes Rev. 2023;24(2):e13534. 36443946 10.1111/obr.13534

[42] Liu C, Wong PY, Tong X, Chow SK, Hung VW, Cheung WH, et al. Muscle plays a more superior role than fat in bone homeostasis: A cross-sectional study of old Asian people. Front Endocrinol (Lausanne). 2022;13:990442. 36714587 10.3389/fendo.2022.990442 PMC9877339

